# Onset of workplace bullying and violence and changes in health-related behaviors: a multi-cohort study

**DOI:** 10.5271/sjweh.4302

**Published:** 2026-09-01

**Authors:** Tianwei Xu, Morten Birkeland Nielsen, Alice J Clark, Reiner Rugulies, Jaana Pentti, Jeppe Karl Sørensen, Mads Nordentoft, Hugo Westerlund, Sari Stenholm, Jussi Vahtera, Ida EH Madsen, Åse Marie Hansen, Marianna Virtanen, Stein Knardahl, Tuula Oksanen, Mika Kivimäki, Linda L Magnusson Hanson, Naja Hulvej Rod

**Affiliations:** 1 Division of Psychobiology and Epidemiology, Department of Psychology, Stockholm University, Stockholm, Sweden.; 2 Department of Public Health, University of Copenhagen, Copenhagen, Denmark.; 3 National Research Centre for the Working Environment, Copenhagen, Denmark.; 4 National Institute of Occupational Health, Oslo, Norway.; 5 Department of Psychosocial Sciences, University of Bergen, Bergen, Norway.; 6 University of Helsinki, Clinicum Unit, Helsinki, Finland.; 7 Finnish Institute of Occupational Health, Helsinki, Finland.; 8 Department of Public Health, University of Turku, Turku, Finland.; 9 Centre for Population Health Research, University of Turku and Turku University Hospital, Turku, Finland.; 10 Research Services, Turku University Hospital and University of Turku, Finland.; 11 National Institute of Public Health, Copenhagen, Denmark.; 12 School of Educational Sciences and Psychology, Philosophical Faculty, University of Eastern Finland, Joensuu, Finland.; 13 Division of Insurance Medicine, Department of Clinical Neuroscience, Karolinska Institutet, Sweden.; 14 School of Medicine, Institute of Public Health and Clinical Nutrition, University of Eastern Finland, Kuopio, Finland.; 15 UCL Brain Sciences, University College London, London, United Kingdom.; 16 Copenhagen Health Complexity Center, University of Copenhagen, Copenhagen, Denmark.

**Keywords:** stress, workplace violence

## Abstract

**Objectives:**

The aim of the study was to examine whether exposure to workplace bullying and violence is associated with changes in health-related behaviors over time.

**Methods:**

This multi-cohort study included four cohorts from Sweden, Denmark, Finland, and Norway, comprising 78 624 participants aged 18–65 years at baseline between 2004 and 2016. The data were analyzed using an emulated trial design. The main analysis ascertained both onset of workplace bullying and violence (exposures) and changes in health-related behaviors (outcome) using data from time T_X_ and T_X+1_ (concurrent analysis). To clarify temporality, changes in health-related behaviors were further calculated at time T_X+1_ to T_X+2_ (longitudinal analysis). We applied logistic regression with generalized estimating equations. Subgroup differences by sex were examined.

**Results:**

Among 125 854 participant-observations across 2–3 study phases of the 78 624 participants, 6–8% experienced onset of workplace bullying and 9–14% reported onset of workplace violence over 1–2 years. The strongest association was observed between onset of violence and becoming obese, with an odds ratio (OR) of 1.13 [95% confidence interval (CI) 1.00–1.27] in the concurrent and 1.31 (95% CI 1.05–1.64) in the longitudinal analysis. In addition, onset of bullying (OR 1.23, 95% CI 1.06–1.44) and violence (OR 1.11, 95% CI 0.99–1.24) were concurrently associated with initiation of excessive alcohol use, with weaker associations in the longitudinal analysis. Exposure–response relationships were observed for all aforementioned associations and findings were consistent across cohorts. Men were more likely to initiate excessive alcohol use than women after experiencing violence (P=0.008).

**Conclusion:**

Exposure to workplace bullying and violence was associated with adverse changes in health-related behaviors.

Workplace bullying and violence are common social stressors with potentially detrimental consequences. Globally, 3–4% of people have reported weekly bullying in a 6-month timeframe (1), while the 12-month prevalence of workplace violence among healthcare workers has been estimated to be as much as 62% (2). Such exposures are linked to higher risks of type 2 diabetes and cardiovascular disease (3, 4), which may be partly through effects on health behaviors. Understanding whether exposure to these psychosocial workplace hazards contributes to changes in health-related behavior may help reduce chronic disease burden in the long term.

Scientific interest in the health consequences of workplace bullying and violence has increased in recent years (5). However, evidence on the potential underlying mechanisms, particularly changes in health-related behaviors, remains limited and inconsistent. The mixed findings largely reflect methodological limitations in research, including the widespread use of cross-sectional study designs (6–11), inadequate control for key confounding variables (6–16), samples restricted to women (14, 15), and occupation-specific settings (12–14, 16, 17) all of which may reduce internal validity and limit the generalizability of results. Furthermore, although reciprocal or feedback mechanisms may exist (18), workplace bullying and violence have commonly been measured at a single, arbitrary time point, typically at study baseline, without considering the timing or onset of exposure. Establishing temporality by examining the onset of exposure in relation to subsequent behavioral changes would considerably strengthen causal inference. In this context, a target trial emulation approach using observational data may provide opportunities to better assess temporality by applying strict eligibility criteria regarding the order of onset of exposure and outcome (19). This could provide insight on how negative changes in health-related behaviors might be prevented under scenarios in which workplace bullying and violence are effectively prevented. To date, no such studies have been published, representing a critical gap in the literature that limits the ability to draw conclusions about causality.

To advance the field, we investigated whether the *onset* of workplace bullying and violence is associated with subsequent changes in health-related behaviors. Specifically, we examined transitions in health behaviors, such as the onset or remission of obesity, initiation or cessation of risky alcohol consumption, smoking relapse or cessation, increases or decreases in physical activity to or from the recommended levels and increase or decrease in the number of behavioral risk factors. This analysis was conducted using longitudinal observational data treated as target trial emulation from four Nordic cohort studies (19).

## Methods

### Study population

**Figure 1:**
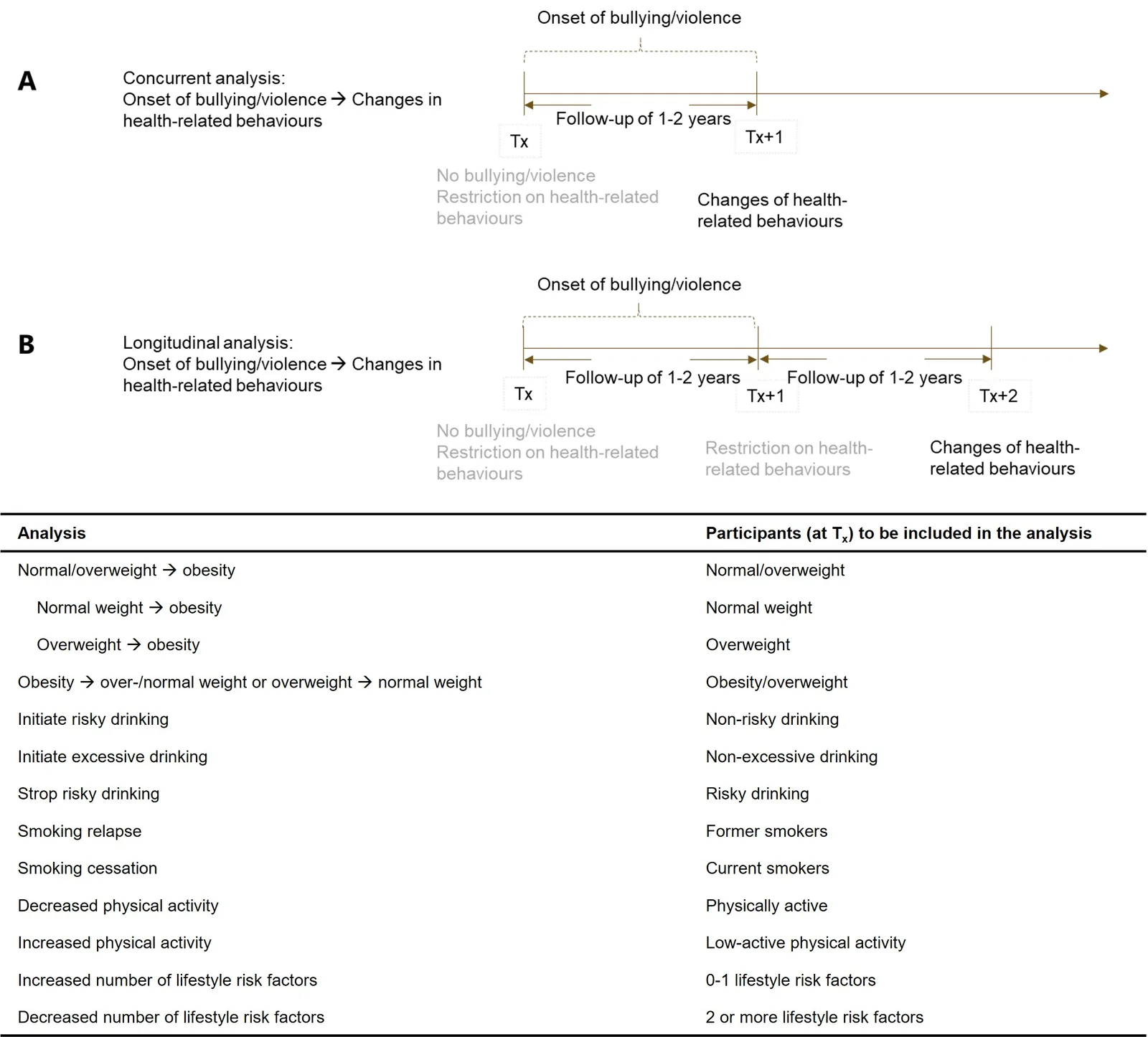
Combined study design of concurrent and longitudinal analyses.

In this multi-cohort study, the study population was drawn from four prospective, biennial cohort studies conducted in the Nordic region: the Swedish Longitudinal Occupational Survey of Health (SLOSH) (20), the Work Environment and Health in Denmark (WEHD) study (21), the Finnish Public Sector (FPS) study (22), and the Norwegian study the New Workplace: Work, Health, and Participation in Working Life (NWP) (18) (table 1; more details in the supplementary material, www.sjweh.fi/article/4302, text S1). We included individuals who participated in at least two consecutive survey waves between 2004 and 2016 (designated T_x_ and T_x+1_, spaced two years apart), were aged 18–65 years at baseline (T_x_), and had complete data on key exposure, outcome, and covariate variables (table 1; figure 1A). This yielded a total of 125 854 participant-observations from 78 624 unique individuals, representing the largest sample available for analysis. Ethical and data access approvals were obtained from the relevant national and institutional review boards: the Regional Ethical Review Board in Stockholm (SLOSH), the Danish Data Protection Agency (WEHD), the Ethics Committee of the Hospital District of Helsinki and Uusimaa (FPS), and the Regional Committees for Medical and Health Research Ethics and the Norwegian Data Inspectorate (NWP) (18, 20, 21).

**Table 1:** Characteristics of participants before and after excluding prevalent cases of exposures and applying restrictions on health-related behaviors. [OB=obesity; OWNW=overweight or normal weight; OWOB=overweight or obesity; RD=risky drinking; NRD=non-risky drinking; PA=physically active; PI=physically inactive; QS=quit smoking; SR=smoking relapse; ≥2 LRF=≥2 lifestyle risk factors; 0–1 LRF=0–1 lifestyle risk factors; SLOSH=Swedish Longitudinal Occupational Survey of Health; WEHD=Work Environment and Health in Denmark study; FPS=Finnish Public Sector study; MWP=the New Workplace: Work, Health, and Participation in Working Life].

		Participant-observations		Participants		Mean age (years)		Women		Born in Nordic countries		Married or cohabiting		Low occupational grade		Poor mental health		Exposure		Outcome of concern		Onset of the outcome
		N		N		SD		N (%)		N (%)		N (%)		N (%)		N (%)		%				%
All observations with ≥2 consecutive waves																						
	Total	125 854		78 624		48		87 171 (69)		12 621 (97)		85 523 (68)		41 705 (33)		16 304 (13)		9/21 *		-		-
	SLOSH	37 329		15 876		50 (9)		21 723 (58)		36 123 (97)		21 342 (57)		12 110 (32)		595 (2)		9/11*				
	WEHD	17 367		10 493		47 (10)		6514 (51)		12 276 (96)		87 56 (68)		47 56 (37)		18 03 (14)		11/10 *				
	FPS	68 863		49 960		47 (10)		54 839 (80)		-		50 500 (73)		21 314 (31)		12 297 (18)		14/31 *				
	NWP	2295		2295		45 (9)		1231 (60)		1984 (96)		1603 (78)		371 (18)		78 (4)		5/6 *				
Emulated-trial population (excluding workplace bullying at T_x_)																						
	Normal or overweight	63 920		44 689		48		64		97		66		30		8		6 **		OB		4
	Obese/overweight	35 873		33 867		49		56		97		66		37		9		6 **		OWNW		11
	Normal weight	37 123		27 001		47		72		97		65		27		8		5 **		OWOB		12
	Overweight	25 090		19 234		49		53		97		67		36		8		6 **		OB		10
	Non-risky drinkers	65 182		49 172		48		70		96		68		32		13		6 **		RD		4
	Risky drinkers	6898		6299		49		22		21		22		10		3		8 **		NRD		42
	Physically inactive	22 257		18 599		49		63		96		66		36		13		6 **		PA		22
	Physically active	58 299		41 716		48		66		97		66		30		7		6 **		PI		39
	Current smokers	10 748		8403		48		65		96		56		46		13		7 **		QS		20
	Former smokers	10 808		9384		48		65		97		74		35		15		6 **		SR		5
	No multiple risk factors	52 476		30 709		47		66		97		66		29		7		5 **		≥2 LRF		11
	With multiple risk factors	18 882		12 185		49		58		97		64		40		13		7 **		0–1 LRF		32
Excluding workplace violence at T_x_																						
	Normal or overweight	75 993		48 518		48		65		97		68		29		10		9 **		OB		4
	Obese/overweight	43 127		37 726		50		57		96		68		36		11		9 **		OWNW		11
	Normal weight	43 918		29 457		47		73		97		67		26		10		10 **		OWOB		11
	Overweight	29 693		22 372		50		54		96		68		35		10		9 **		OB		9
	Non-risky drinkers	79 098		53 685		48		68		97		68		31		11		10 **		RD		4
	Risky drinkers	9111		7747		49		61		98		67		31		18		10 **		NRD		43
	Physically inactive	28 417		22 349		49		65		96		68		36		15		9 **		PA		22
	Physically active	67 812		45 337		48		67		97		67		29		9		10 **		PI		39
	Current smokers	12 390		9054		49		64		96		57		45		15		10 **		QS		20
	Former smokers	13 628		10 706		49		66		97		74		34		17		12 **		SR		6
	No multiple risk factors	61 482		42 976		48		68		97		68		27		9		10 **		≥2 LRF		12
	With multiple risk factors	17 861		19 236		50		60		96		66		39		15		14 **		0–1 LRF		30

* Prevalence of bullying and prevalence of violence. ** Incidence proportion.

### Study design

We emulated a target trial using observational data from two (concurrent analysis) and three (longitudinal analysis) consecutive survey waves (19). The specification of target trials and emulations using observational data are presented in supplementary table S1. In the concurrent analysis (based on 6898–79 098 participant-observations), participants were included if, at baseline (T_x_), they met the following criteria: (i) were not exposed to workplace bullying in bullying-related analyses or to violence in violence-related analyses; and (ii) did not exhibit the specific health-related behavior outcome under investigation (table 1; figure 1A).

For the longitudinal analysis (based on 1069–32 267 participant-observations), we applied more stringent eligibility criteria to ensure that changes in health-related behaviors occurred *after* the onset of workplace bullying or violence. In addition to the baseline restrictions used in the concurrent analysis [criteria (i) and (ii) at T_x_], criterion (ii) was also required to be met at T_x+1_. This ensured that any behavioral changes observed between T_x+1_ and T_x+2_ followed the onset of exposure between T_x_ and T_x+1_ (figure 1B).

### Assessment of workplace bullying and violence

In all four cohorts, workplace bullying and workplace violence, including physical violence and threats of violence, were assessed via self-administered questionnaires. Participants were asked whether they had experienced bullying or violence within a specified recall period of the past 6, 12, or 24 months (supplementary tables S2 and S3).

In the SLOSH and WEHD cohorts, additional data on the frequency of exposure were collected. Based on this information, participants in these cohorts were categorized into three exposure groups: (i) frequently exposed (exposure reported on a weekly or daily basis), (ii) occasionally exposed (exposure reported less frequently than weekly), and (iii) not exposed. This categorization enabled the investigation of potential exposure–response relationships.

### Health-related behaviors

Body mass index (BMI) was calculated from self-reported weight and height (kg/m^2^). Following World Health Organization classification criteria, participants were categorized as underweight (<18.5 kg/m^2^), normal weight (18.5–24.9 kg/m^2^), overweight (25.0–29.9 kg/m^2^), or obese (≥30.0 kg/m^2^). For the main analyses, weight gain was defined as transitioning from normal or overweight to obesity, and weight loss as transitioning from obese to overweight/normal or from overweight to normal. Additional transitions, such as from normal to overweight/obese and from overweight to obese, were analyzed in supplementary analyses.

Alcohol use was classified as risky or non-risky based on sex-specific thresholds from earlier Danish and Finnish guidelines: >14 units/week for women and >21 units/week for men (22). Risk classification was determined using the Alcohol Use Disorders Identification Test (AUDIT) in SLOSH (2006/2008), WEHD, and FPS, and the CAGE questionnaire (Cutting down, Annoyance by criticism, Guilty feelings, and Eye-openers) in SLOSH (2010–2016) (Supplementary Text S2). Comparable data on alcohol consumption were not available for the NWP cohort. Initiation of risky drinking was defined as a transition from non-risky to risky consumption, while cessation referred to a reduction from risky to non-risky levels.

Participants were classified as having low physical activity if they reported never or rarely engaging in physical activity (SLOSH) or reported ≤14 metabolic equivalent task (MET) hours per week (WEHD, FPS, and NWP) (23). Changes in physical activity were categorized as increased activity (transition from low to moderate/high activity) or decreased activity (transition from moderate/high to low).

Current smoking was defined as a current smoker of ≥1 cigarette per day (SLOSH, FPS, WEHD and NWP). Former smokers were those who reported they used to be smokers but did not smoke anymore (FPS and WEHD). Smoking cessation referred to quitting, while relapse meant resuming smoking (≥1 cigarette/day).

Additionally, we created a variable indicating the existence of multiple risky lifestyle factors (0–1 versus ≥2 risky factors), in SLOSH, WEHD and FPS, where information on BMI, alcohol, smoking and physical activity was available.

### Covariates

Key confounders were identified using directed acyclic graphs, and included age, sex (men/women), occupational grade (low, medium, high), country of birth (Nordic, other European, other), marital status (married or cohabiting, single, divorced or separated, widowed), and self-reported current mental health problems (yes/no). Additional potential confounders included night shift (yes/no) and sleep disturbances (yes/no), but were adjusted in separate steps. All covariates were obtained through linkage with national register data in Sweden, Denmark, Finland, and Norway. The exceptions were marital status in FPS, and mental health symptoms, shift work, and sleep disturbances, which were self-reported in all other cohorts. In the FPS cohort, information on country of birth was not available; however, the vast majority of participants were born in Nordic countries. Mental health symptoms were assessed using self-reported questionnaires and quantified as a continuous measure of depressive symptoms at baseline (T_x_) in all cohorts. Additional details on the health-related behaviors and covariates are provided in supplementary text S2. All covariates were measured at baseline (T_x_).

### Statistical analysis

In the main analyses, we adjusted for key confounders, which were measured at baseline, including sex, country of birth, baseline year, and age, occupational grade, marital status, and mental health symptoms. Because the exposure (onset of workplace bullying or violence) and the outcome (change in health-related behavior) were assessed between baseline (T_x_) and a follow-up wave (T_x+1_ or T_x+2_), all covariates (measured at T_x_) were assumed to temporally precede both exposure and outcome. Each exposure–outcome pair was analyzed separately. To strengthen causal inference, we assessed exposure–response relationships in the SLOSH and WEHD cohorts, where frequency of exposure was available. These were modelled under the assumption of monotonicity (ie, increasing exposure associated with increasing risk of behavior change). To reduce potential heterogeneity due to differences in recall periods across cohorts, we conducted a sensitivity analysis restricted to survey waves in which bullying and violence were assessed using a consistent 12-month reference timeframe. Moreover, to address other potential baseline confounders such as nightshift work and sleep disturbances, these factors were adjusted in the supplementary analyses together with the key confounders. Sex-stratified analyses were also performed, and potential sex differences were evaluated using multiplicative interaction terms.

Binomial logistic regressions were conducted. We applied the framework of generalized estimating equations, as some participants may contribute to several different T_x_. All analyses were conducted separately for each cohort using SAS 9.4 (Proc Logistic or Proc Genmod). The cohort-specific estimates were combined using fixed-effect meta-analyses (24), performed in the R package “meta” version 4.8-4.

## Results

### Baseline characteristics

Of 78 624 participants (125 854 observations), 9% reported workplace bullying and 21% workplace violence at baseline (T_x_). Among those unexposed at T_x_, incidence at T_x+1_ was 6–8% for bullying and 9–12% for violence (table 1; supplementary table S4).

In WEHD (N=17 375), bullying was mainly perpetrated by colleagues, supervisors, or subordinates, whereas workplace violence was more often committed by external parties such as customers, clients, patients, or students (supplementary figure S1).

Outcome-specific baseline characteristics of the study population are summarized in table 1.

### Body mass index categories

**Figure 2:**
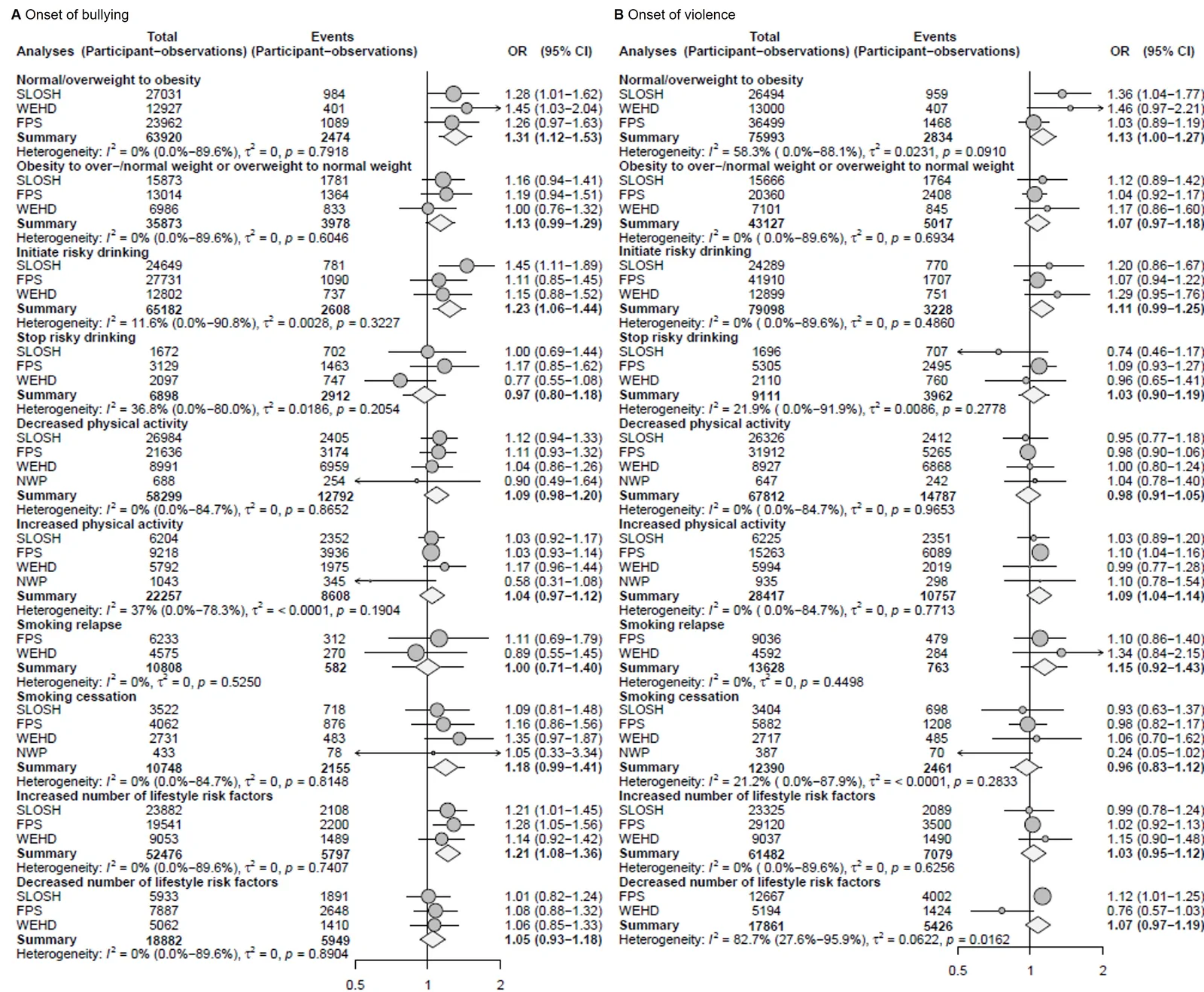
Onset of workplace bullying (A), onset of workplace violence (B) and changes in health-related behaviors in concurrent analysis with adjustment for age, sex, marital status, country of birth, occupational grade and mental health.

**Figure 3:**
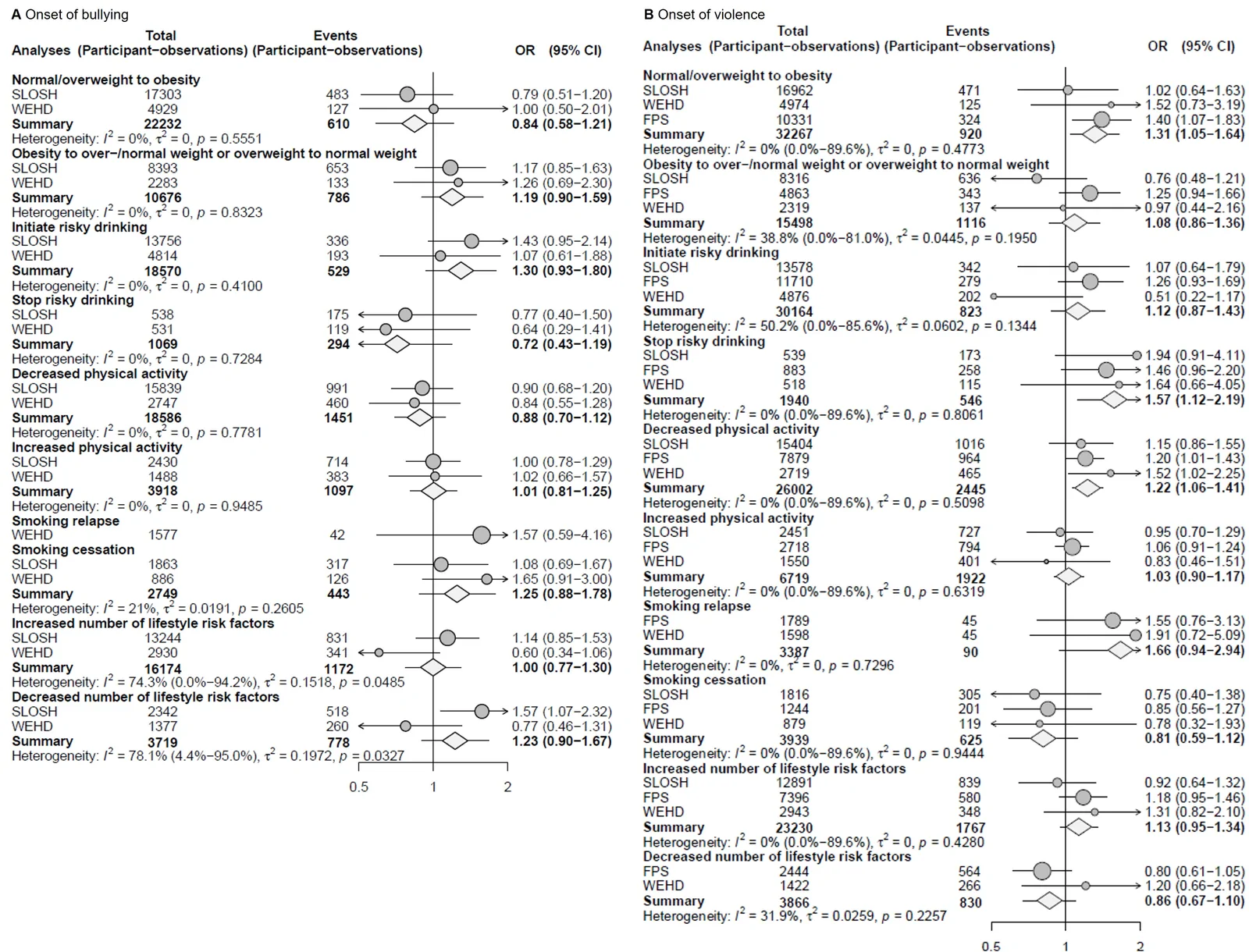
Onset of workplace bullying (A), onset of workplace violence (B) and changes in health-related behaviors in longitudinal analysis with adjustment of age, sex, marital status, country of birth, occupational grade and mental health.

In the fully-adjusted concurrent analysis (figure 2), onset of workplace bullying was associated with higher odds of gaining from normal/overweight to obese [OR 1.31, 95% confidence interval (CI) 1.12–1.53], which was mainly explained by the weight gain from overweight to obesity (OR 1.19, 95% CI 1.01–1.41) (supplementary figure S2 and S3). However, all these associations were not replicated in the longitudinal analysis (figure 3).

A similar association was observed for onset of workplace violence and becoming obese (OR 1.13; 95% CI 1.00–1.27) (figure 2). Longitudinal analyses confirmed this association (OR 1.31, 95% CI 1.05–1.64) (figure 3).

Neither onset of bullying nor onset of violence was associated with weight loss, ie, changing from obesity to overweight/normal weight or from overweight to normal weight (figures 2 and 3).

**Figure 4:**
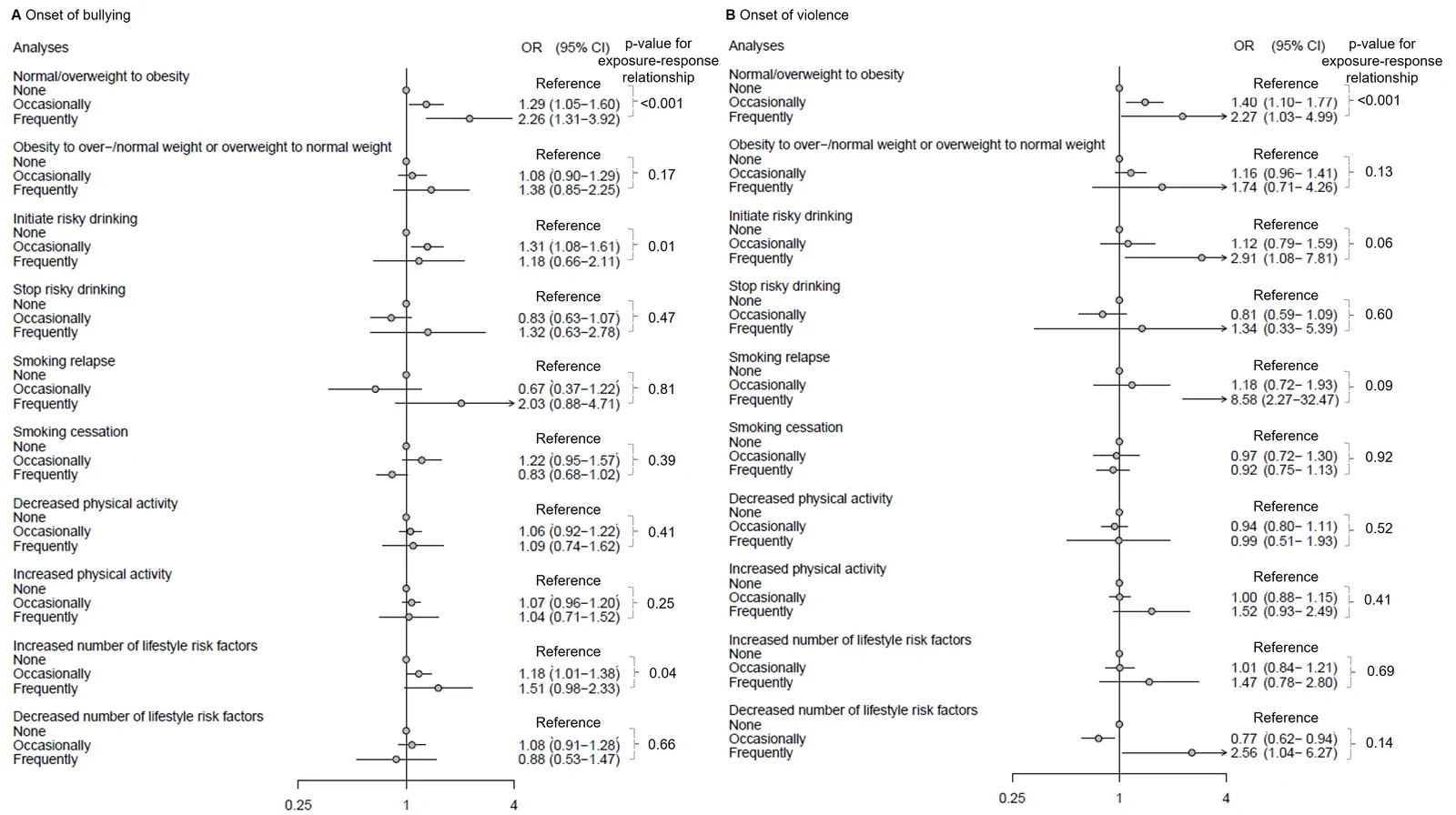
Exposure–response relationship of onset of workplace bullying (A) and onset of workplace violence (B) on health-related behavioral changes in SLOSH and WEHD. None=less than once a year; Occasionally=at least once in a year; Frequently=daily or weekly.

Exposure–response relationships were observed between the frequency of bullying or violence and the likelihood of becoming obese (P<0.001), but no such trend was observed for weight loss (figure 4).

### Alcohol intake

The onset of workplace bullying was associated with an increased likelihood of initiating risky drinking in the concurrent analysis (OR 1.23; 95% CI 1.06–1.44) (figure 2), showing a statistically significant exposure–response relationship (P=0.01) (figure 4). A similar but weaker association was observed for the onset of workplace violence (OR 1.11, 95% CI 0.99–1.24) (figure 2), with an exposure–response trend that approached statistical significance (P=0.06) (figure 4). In the longitudinal analyses, point estimates were comparable but did not reach statistical significance for either exposure (figure 3).

In the concurrent analyses, neither the onset of bullying nor violence was associated with cessation of risky drinking (figure 2). However, a significant association emerged in the longitudinal analysis: participants who reported risky drinking at both T_x_ and T_x+1_ had higher odds of stopping risky drinking at T_x+2_ if they experienced the onset of workplace violence (OR 1.57, 95% CI 1.12–2.19) (figure 3).

### Smoking behaviors

We did not find any clear associations between onset of bullying or violence and changes in smoking behaviors (figures 2–4). However, there was a suggestive concurrent association between the onset of bullying and increased odds of smoking cessation (OR 1.18, 95% CI 0.99–1.41) (figure 2). Although this result did not reach statistical significance, all four cohort-specific estimates pointed in the same direction (figure 2).

### Physical activity

The onset of workplace bullying was not associated with changes in physical activity levels in either the concurrent or longitudinal analyses (figures 2–4). Conversely, the onset of workplace violence showed divergent associations across timeframes. In the concurrent analysis, exposure to violence was associated with increased physical activity (OR 1.09, 95% CI 1.04–1.14), whereas in the longitudinal analysis, it was associated with a reduction in physical activity (OR 1.22, 95% CI 1.06–1.41) (figures 2 and 3). No clear exposure–response relationships were observed for either direction of change (figure 4).

### Number of lifestyle risk behaviors

There was a concurrent association between onset of workplace bullying and increased number of risky lifestyle factors (OR 1.21, 95% CI 1.08–1.36) (figure 2), with exposure–response trends (P=0.04) (figure 4), which was not shown in the longitudinal analysis (figure 3). We did not observe a similar tendency for workplace violence (figure 2–4).

### Sensitivity analysis

Pooling only the data with a 12-month timeframe showed similar results as in the main analysis (supplementary figure S4). Additional adjustments for baseline nightshift work and sleep disturbances in the model did not materially change the results (supplementary figures S5-10).

In sex-stratified analyses, the onset of workplace bullying among women was associated with both an increased likelihood of becoming obese (OR 1.40, 95% CI 1.17–1.68), with obesity remission (OR 1.21, 95% CI 1.05–1.39) and an increased number of risky lifestyle factors (OR 1.34, 95% CI 1.18–1.54). Among men, the onset of bullying was significantly associated with smoking cessation (OR 1.46, 95% CI 1.15–1.86). However, no statistically significant sex differences in these associations were observed on the multiplicative interaction scale (supplementary figure S11).

In terms of the onset of exposure to violence, men exposed to violence were more likely to initiate risky drinking (OR 1.49, 95% CI 1.23–1.82), whereas no such association was observed among women (OR 0.99, 95% CI 0.87-1.13), with a significant interaction by sex (P=0.001) (supplementary figure S11). There was also a suggestion that women may be more likely than men to relapse into smoking following exposure to workplace violence (women: OR 1.30, 95% CI 1.02–1.66; men: OR 0.76, 95% CI 0.46–1.26; P for sex interaction=0.06) (supplementary figure S11).

## Discussion

Using longitudinal data from four Nordic population-based cohorts, we emulated a non-randomized intervention study to examine the impact of workplace bullying and violence on changes in health-related behaviors. Our findings add to the growing body of evidence indicating that these psychosocial stressors may play a significant role in the development of adverse health behaviors. By focusing on the onset of exposure, we strengthen the causal interpretation of previous associations and demonstrate consistent findings across both concurrent and longitudinal timeframes, as well as across diverse national cohorts. The most robust evidence emerged for the association between the onset of workplace violence and subsequent development of obesity, followed by weaker associations between the onset of bullying/violence and initiation of excessive alcohol consumption, in which the longitudinal associations were only suggestive. These findings underscore the long-term health risks of workplace bullying and violence and the need for preventive and intervention strategies in occupational settings.

Several theoretical models highlight changes in health-related behaviors as coping strategies when facing stressful situations (22, 25, 26). Energy-dense food and alcohol are easily accessible and may be used to alleviate perceived distress (22, 26, 27). Potential mechanisms include changes in reward circuitry and co-activated brain systems such as the hypothalamic-pituitary-adrenal axis, and signaling through many neurotransmitters, eg, norepinephrine, corticotropin-releasing factor and dynorphin (25, 26). However, eating energy-dense food and consuming alcohol to cope with chronic distress are generally considered to be maladaptive (22, 25, 26), as they may contribute to the development of chronic diseases.

To our knowledge, this is the first longitudinal study on the onset of workplace violence and obesity, consistent with evidence linking childhood exposure to violence with obesity risk (28, 29). We found only concurrent, not longitudinal, associations between bullying onset and weight change, which contrasts with prior findings of a subsequent modest BMI change of 1-unit or more after experiencing onset of workplace bullying (30). Nevertheless, even modest weight gain when occurring together with workplace bullying may be associated with a more than 4 times higher risk of incident type 2 diabetes (30). Stressful events may contribute to chronic disease through short-term weight gain and/or long-term obesity, warranting further research on underlying mechanisms.

Our finding on onset of workplace bullying, violence and uptake of excessive drinking corroborates results from previous cross-sectional studies and longitudinal studies with single measures of exposure (6, 10, 17). We added to this literature by providing evidence on exposure–response relationship and examining the relationship between *onset* of bullying and violence rather than assessing their effect at a random time point. While our longitudinal analyses showed similar direction as in the concurrent analyses, the magnitude of the effects were reduced.

Unlike previous studies showing a correlation between workplace harassment and higher prevalence of smoking (6), our study showed a tendency for men, but not women, to quit smoking after being exposed to workplace bullying. One previous empirical study suggested that the motivation of quitting smoking may be increased due to the disapproval of smoking by others and the feeling of shame, particularly among men (25). Other speculations of the findings may include that bullying occurred in social situations, such as during smoking pauses.

We found no association between onset of bullying and increases/decreases in physical activity, which contrasts with an Australian study that reported increased activity three years after workplace bullying exposure among women (15). The same study found decreased activity three years after experiencing general physical violence, in agreement with our findings (15). Surprisingly, in the concurrent analysis of our study, previously inactive employees became more active while experiencing onset of workplace violence. Further research is needed to clarify causation.

We only observed increased number of risky lifestyle factors immediately after experiencing onset of bullying, but not violence. A previous study reported that only 2–4% of individuals experienced simultaneous exposure to both workplace bullying and violence or threats of violence (3), suggesting that these are largely distinct psychosocial stressors. Workplace violence is typically perpetrated by external parties such as clients, customers, or patients, whereas bullying tends to originate from within the organization, involving co-workers, subordinates, or supervisors. While violence often reflects isolated but severe incidents, bullying is usually characterized by repeated, persistent exposure over time (3, 4). The emotional consequences of these stressors appear to differ depending on the nature of the exposure (eg, acuteness and duration) (2), potentially influencing behavioral outcomes through distinct motivational and coping mechanisms (31).

Interestingly, we observed some indications of positive health-related behavioral changes following exposure. For example, women who experienced bullying were more likely to lose weight, men were more likely to quit smoking after bullying, and individuals increased their physical activity or stopped risky drinking following experiences of workplace violence. These changes varied depending on the type of stressor and the timing of assessment. Importantly, these findings should be interpreted with caution, as they may reflect chance variation and/or healthy worker effect, where those who were bullied were more likely to select out of work, and therefore are not part of the follow-up questionnaires (32). The lack of consistency across both concurrent and longitudinal analyses and the absence of exposure–response relationships limit the confidence regarding causality. Nevertheless, recent literature has suggested the potential of post-traumatic growth, ie, the positive changes that can arise from the struggle to cope with the severely stressful situation (33). This perspective underscores the importance of better understanding the dynamics and attributes of health-related behavioral changes. Such insights may inform the development of targeted interventions, particularly with regard to critical time windows and the motivational pathways underlying both adaptive and maladaptive responses to workplace stressors.

Potential sex differences may exist in responses to workplace violence, which may imply differences in reinforcement mechanisms due to differences in the critical structures and neurotransmitters between men and women (25, 26). For instance, female former smokers were more likely to relapse than men after exposure to violence, consistent with prior findings (14). Stress may heighten smoking cravings more in women, who may experience greater relief upon resuming smoking after abstinence (25). Conversely, we observed workplace violence being linked to excessive alcohol intake, particularly in men. These sex-specific patterns underscore the need for increasing awareness of stress-related behavioral changes, which may differ between men and women.

### Strengths and limitations

To ensure temporality while simultaneously elucidating the short-term effects of bullying/violence, we presented results from two analytical designs (concurrent and longitudinal). The large sample size enabled both concurrent and exposure–response analyses, providing robust statistical power for emulating target trials using observational studies.

However, some limitations in statistical power persisted for the more restrictive longitudinal design, especially for NWP due to its smaller sample size. Moreover, the use of “emulated trial” terminology might unintentionally suggest a level of comparability with randomized trials. The causality of our findings should thus be carefully discussed. For example, as workplace bullying and violence were measured using self-report, exposure misclassification could occur due to varying measurement timeframes (1). However, restricting the analyses to study waves with the same timeframe only had minor impact on the results. Health-related behaviors were assessed through self-reports. The results should therefore be interpreted with caution, as the association between the onset of bullying/violence and changes in health-related behaviors may be slightly under- or overestimated if individuals exposed to bullying or violence, for example, were more likely to underreport weight or overreport physical activity than the unexposed. While these behaviors are likely to suffer from some misclassification, we do not expect large differences between groups. Also, while we were not able to distinguish between people who experienced an onset of bullying/violence just at their middle age and those who experienced repeated exposure across life stages, one should be cautious to interpret the findings. Unmeasured confounding, such as personality traits, genetic predispositions, concurrent life events, and other aspects of mental health beyond symptoms, may have influenced the observed associations. We addressed this by adjusting for baseline depressive symptoms, which may have removed the confounded effects of these factors on both workplace bullying/violence reporting and health behavior changes to some extent. Finally, although not all four cohorts were nationally representative, the results remained consistent across cohorts with a low level of heterogeneity, enhancing the generalizability of our findings. Nevertheless, one should be cautious when extrapolating our findings to vastly different cultural settings and societies dissimilar to the Nordic countries.

In conclusion, onset of workplace bullying and workplace violence may contribute to changes in health-related behaviors, especially increasing the risk of becoming obese after the exposure to onset of workplace violence. With over 10% of employees reporting onset of such experiences, addressing these behaviors is crucial for health promotion. Further research is needed to evaluate the health benefits of interventions targeting workplace bullying and violence.

## Supplementary material

Supplementary material

## Data Availability

The data used in this study are from the SLOSH study, the WEHD study, the FPS study, and the NWP study. The access of these datasets is restricted to approved researchers. Access to the SLOSH data may be provided for research in line with Swedish law, and data requests should be sent to data@slosh.se. The WEHD study is based on anonymized microdata available from Statistics Denmark. Access to data can only be permitted through an affiliation with a Danish authorized environment. Researchers interested in collaboration should contact the National Research Centre for the Working Environment, Copenhagen, Denmark (email: nfa@nfa.dk, contact persons: Dr Jeppe Karl Sørensen and Prof. Reiner Rugulies). In FPS, pseudonymized questionnaire data as used in this study can be shared by request to FIOH scientific committee (Dr Jenni Ervasti, jenni.ervasti@ttl.fi). The NWP data presented in this article are not readily available because the license to collect and store the data from the Data Inspectorate and REK stated specifically that de-identified data will be available only to collaborators with the National Institute of Occupational Health (NIOH) in Norway upon signing a declaration of confidentiality. Data are not publicly available due to the terms of participation.Funding
