# Supplementary material for: Onset of workplace bullying and violence and changes in health-related behaviors: a multi-cohort study
Source: Scand J Work Environ Health. 2026 Aug 31;52(5):549–59. doi: 10.5271/sjweh.4302 (PMC13550778; doi:10.5271/sjweh.4302)

Contents

Supplementary Text S1. Cohorts included in the study 2

Supplementary Text S2. Additional details of measurement of health-related behaviors and covariates. 3

Supplementary Table S1. Specification of target trials and emulations using observational data. 4

Supplementary Table S2. Measurements of workplace bullying 5

Supplementary Table S3. Measurements of workplace violence 6

Supplementary Table S4. Characteristics of participants for analyses of categories of body mass index. 7

Supplementary Figure S1. Distribution of perpetrator of workplace bullying and violence in the Work Environment and Health in Denmark study (N=17,375). 8

Supplementary Figure S2. Onset of workplace bullying (A and C), onset of workplace violence (B and D) and changes in categories of body mass index. 9

Supplementary Figure S3. Exposure-response analysis of the concurrent association between onset of workplace bullying (A), violence (B) and changes of categories of body mass index. 10

Supplementary Figure S4. Onset of workplace bullying (A), onset of workplace violence (B) and changes in health-related behaviours pooled only in data using 12 months' timeframe in the exposure measurement. 11

Supplementary Figure S5. Onset of workplace bullying (A), onset of workplace violence (B) and changes in health-related behaviours in concurrent analysis, with additional adjustment on nightshift work. 12

Supplementary Figure S6. Onset of workplace bullying (A), onset of workplace violence (B) and changes in health-related behaviours in concurrent analysis, with additional adjustment on nightshift work and sleep disturbances. 13

Supplementary Figure S7. Onset of workplace bullying (A), onset of workplace violence (B) and changes in health-related behaviours in longitudinal analysis, with additional adjustment on nightshift work. 14

Supplementary Figure S8. Onset of workplace bullying (A), onset of workplace violence (B) and changes in health-related behaviours in longitudinal analysis, with additional adjustment on nightshift work and sleep disturbances. 15

Supplementary Figure S9. Exposure-response relationship of onset of workplace bullying (A) and onset of workplace violence (B) on health-related behavioural changes in SLOSH and WEHD. None=less than once a year; Occasionally=at least once in a year; Frequently=daily; weekly., with additional adjustment on nightshift work. 16

Supplementary Figure S10. Exposure-response relationship of onset of workplace bullying (A) and onset of workplace violence (B) on health-related behavioural changes in SLOSH and WEHD. None=less than once a year; Occasionally=at least once in a year; Frequently=daily; weekly., with additional adjustment on nightshift work and sleep disturbances. 17

Supplementary Figure S11. Onset of workplace bullying (A), onset of workplace violence (B) and changes in health-related behaviours stratified by sex. 18

Supplementary Text S1. Cohorts included in the study

Swedish Longitudinal Occupational Survey of Health (SLOSH) started with the first wave in 2006 with those who responded to Swedish Work Environment Study (SWES) in 2003 (1). For the later waves in 2008 (response proportion: 61%), 2010 (response proportion: 57%), 2012 (response proportion: 57%), 2014 (response proportion: 53%), respondents to SWES 2005, 2007, 2009 and 2011 were added into each wave respectively for further follow-up visits. After 2014, no new participants were added and the response portion in 2016 for those participate in 2014 was 51%. In our study, we used SLOSH participants of waves 2010, 2012, 2014, and 2016, who aged between 18 and 65 and were gainfully employed at the time of participation.

The Working Environment and Health Denmark (WEHD) is a cohort based on biannual surveys conducted from a random sample of the Danish work force, aged 18 to 65 (2). WEHD consists of a series of cross-sectional random samples of the Danish work force, extracted in 2012, 2014, 2016, and 2018. Approximately 35,000 workers were approached, and the respond rates were around 50%. Respondents from the 2012 random sample were invited to answer the questionnaire in 2014 and 2016 as well. Among the eligible respondents from the 2012 random sample 72.4% responded in 2014, 68.3% responded in 2016.

Finnish Public Sector study (FPS) is an open cohort established in 1997-1998 (3). It consists of employees in the municipal services of 10 Finnish towns and 21 public hospitals, aged between 18 and 65 years with a job contract for a minimum of 6 months in their employers' records.

Data for workplace bullying: 10Town-surveys in 2014 (N=62769, 68.6%) and 2016 (N=65089, 71.5%).

Data for workplace violence: 10Town-surveys in 2012 (N=39250, 69.0%), 2014, 2016.

'The new workplace: Work, health, and participation in the new work life' study (NWP) is a cohort based on 48 Norwegian organizations (31 private and 17 public), recruited using the convenience sampling (N=12603).[21] Baseline was created from 2004 to 2009 (N=6097, response proportion: 48.4%) and the baseline participants was followed two years later from 2006 to 2011.[21] A total of 2295 participants were involved with the participation in both waves (response proportion: 37.6%).

Supplementary Text S2. Additional details of measurement of health-related behaviors and covariates.

Information on alcohol consumption: calculated using the sum of the average of response categories as an aggregated measure for a typical week in FPS and WEHD using Alcohol Use Disorders Identification Test (AUDIT). In SLOSH, alcoholism was identified if participants had a score of 2 or more, according to the Cutting down, Annoyance by criticism, Guilty feeling, and Eye-openers (CAGE).

Metabolic equivalent of task hours of physical activity: calculated based on the average time spent on physical activities with different intensity levels as listed in the questionnaire.

Occupational grade was coded according to the national-specific ISCO-code and characterized into “high” (e.g., managers and professionals), “intermediate” (e.g., technicians and associate professionals), and “low” (e.g., service- and shop sales workers and machine operators).

Depressive symptoms were measured using a six-item version of the symptom checklist in SLOSH,[27] and the Major Depression Inventory in WEHD,[28] two screening questions in FPS[29] and one question in NWP.[19]

Sleep disturbances were defined as self-reported difficulties in initiating or maintaining sleep, non-restorative sleep, or daytime tiredness. These were assessed over the past 4 weeks in WEHD and FPS, and over the past 3 months in SLOSH, using the Karolinska Sleep Questionnaire (3 or 4 items in WEHD and SLOSH) or the Jenkins Sleep Problem Scale (4 items in FPS). Sleep disturbances were considered present if respondents reported any of these symptoms at least “often” or “all the time” in WEHD, 2-4 nights per week in FPS, or 3-4 nights per week in SLOSH.

Nightshift work was assessed by self-reports (SLOSH, FPS and WEHD).

Abbreviation:

FPS=Finnish Public Sector study

WEHD=Working Environment and Health in Denmark study

SLOSH=Swedish Longitudinal Occupational Survey of Health

NWP= Norwegian ‘The new workplace: Work, health, and participation in the new work life’ study

Supplementary Table S1. Specification of target trials and emulations using observational data.

Component	Target trial	Target trial emulation
Eligibility criteria	Aged between 18 and 65 at baseline;<br>Employed at baseline;<br>Depending on the outcome of concern, participants may also be:<br>Normal; overweight;<br>Obese/overweight;<br>Normal weight;<br>Overweight;<br>Non-risky drinkers;<br>Risky drinkers;<br>Physically inactive;<br>Physically active;<br>Current smokers;<br>Former smokers;<br>No multiple risk factors;<br>With multiple risk factors;	Participated in at least two consecutive survey waves between 2004 and 2016;<br>Aged between 18 and 65 at baseline;<br>Employed at baseline;<br>Had complete data on key exposure, outcome, and covariate variables;<br>Depending on the outcome of concern, participants may also be:<br>Normal; overweight;<br>Obese/overweight;<br>Normal weight;<br>Overweight;<br>Non-risky drinkers;<br>Risky drinkers;<br>Physically inactive;<br>Physically active;<br>Current smokers;<br>Former smokers;<br>No multiple risk factors;<br>With multiple risk factors.
Treatment strategies	For workplace bullying:<br>Onset of exposure to workplace bullying vs. remain unexposed to workplace bullying.<br><br>For workplace violence:<br>Onset of exposure to workplace violence vs. remain unexposed to workplace violence.	Same as in target trial.
Treatment assignment	Eligible persons will be randomly assigned to one strategy and will be aware of which strategy they were assign to.	Eligible persons will be assigned to the strategies with which their data were compatible at the time of eligibility.
Outcomes	The corresponding outcomes for different eligible participants are:<br>Obesity;<br>Overweight; normal weight; Overweight; obesity; Risky drinking;<br>Non-risky drinking;<br>Physically active;<br>Physically inactive;<br>Quit smoking;<br>Smoking relapse;<br>≥2 lifestyle risk factors;<br>0 – 1 lifestyle risk factor.	Same as in target trial.
Follow-up	Immediately after the treatment assignment (concurrent);<br>Two years after the treatment assignment (longitudinal).	Same as in target trial.
Causal estimand	Intention-to-treat effect;<br>Per-protocol effect.	Observational analogue of the per-protocol effect.
Statistical analysis	Intention-to-treat analysis;<br>Per-protocol analysis.	Same as per-protocol analysis, with the adjustment of pre-assignment confounders.

Supplementary Table S2. Measurements of workplace bullying

Questions on workplace bullying victimisation	Cohort baselines	Timeframe at baseline	First follow-up	Timeframe at first follow-up
Are you subjected to personal persecution (in Swedish: 'personlig förföljelse') in the form of unkind words; behaviour from your superiors; fellow workers?	SLOSH 2006	12 months	SLOSH 2008	12 months
	SLOSH 2008	12 months	SLOSH 2010	12 months
	SLOSH 2010	12 months	SLOSH 2012	6 months
	SLOSH 2012	6 months	SLOSH 2014	6 months
	SLOSH 2014	6 months	SLOSH 2016	6 months
	SLOSH 2016	6 months	SLOSH 2018	6 months
Have you been subjected to workplace bullying in the last 12 months? (The action was conducted by one; more people regularly and over a long period of time; in a repeated manner.)	WEHD 2012	12 months	WEHD 2014	12 months
	WEHD 2014	12 months	WEHD 2016	12 months
Have you been subjected to such bullying? (Workplace bullying refers to a situation where someone is subjected to social isolation; exclusion, his; her work and efforts are devalued, he; she is threatened, derogatory comments are made about him; her in his; her absence,; other negative behaviour that aims to torment, wear down,; frustrate the victim occurs.)	FPS 2014	12 months	FPS 2016	12 months
Have you been subjected to bullying; harassment in the workplace during the last six months? (To be labelled as bullying at work, the offensive behaviour has to occur repeatedly over a period of time, and the person confronted has to experience difficulties defending himself/herself. The behaviour is not bullying if two parties of approximately equal 'strength' are in conflict; the incident is an isolated event.)	NWP 2004	6 months	NWP 2006	6 months
	NWP 2005	6 months	NWP 2007	6 months
	NWP 2006	6 months	NWP 2008	6 months
	NWP 2007	6 months	NWP 2009	6 months
	NWP 2008	6 months	NWP 2010	6 months
	NWP 2009	6 months	NWP 2011	6 months

Supplementary Table S3. Measurements of workplace violence

Questions on workplace violence	Cohort baselines	Timeframe at baseline	First follow-up	Timeframe at first follow-up
Are you exposed to violence; threats of violence at work?	SLOSH 2006	12 months	SLOSH 2008	12 months
	SLOSH 2008	12 months	SLOSH 2010	12 months
	SLOSH 2010	12 months	SLOSH 2012	6 months
	SLOSH 2012	6 months	SLOSH 2014	6 months
	SLOSH 2014	6 months	SLOSH 2016	6 months
	SLOSH 2016	6 months	SLOSH 2018	6 months
In the last 12 months, have you been subjected to threats of violence at your work place? In the last 12 months, have you been subjected to physical violence at your work place? (Answering 'yes' to at least one of the two questions)	WEHD 2012	12 months	WEHD 2014	12 months
	WEHD 2014	12 months	WEHD 2016	12 months
Have you been exposed to violence; threats at work in the last 12 months? (Answering 'yes' to 'objects thrown around, premises smashed', 'psychological violence (e.g., verbal threats)', 'physical violence (e.g. punches and kicks)'; 'threatened with weapon (e.g. a gun)')	FPS 2012	12 months	FPS 2014	12 months
	FPS 2014	12 months	FPS 2016	12 months
Have you been exposed to threats; violence at work during the last two years? (Answering if 'seldom', 'sometimes', 'often', 'very often'; 'always')	NWP 2004	24 months	NWP 2006	24 months
	NWP 2005	24 months	NWP 2007	24 months
	NWP 2006	24 months	NWP 2008	24 months
	NWP 2007	24 months	NWP 2009	24 months
	NWP 2008	24 months	NWP 2010	24 months
	NWP 2009	24 months	NWP 2011	24 months

Supplementary Table S4. Characteristics of participants for analyses of categories of body mass index.

	Participant-observations, (N)	Participants, (N)	Mean age, years (SD)	Women, n (%)	Born in Nordic countries, n (%)	Married; cohabiting, n (%)	Low occupational grade, n (%)	Poor mental health, n (%)	Exposure, (%)	Outcome of concern	Onset of the outcome, (%)
Excluding workplace bullying at Tx											
Normal; overweight	63920	44689	48	64	97	66	30	8	6**	Obesity	4
Obese/overweight	35873	33867	49	56	97	66	37	9	6**	Overweight; normal weight	11
Normal weight	37123	27001	47	72	97	65	27	8	5**	Overweigh; /obesity	12
Overweight	25090	19234	49	53	97	67	36	8	6**	Obesity	10
Excluding workplace violence at Tx											
Normal; overweight	75993	48518	48	65	97	68	29	10	9**	Obesity	4
Obese/overweight	43127	37726	50	57	96	68	36	11	9**	Overweight; normal weight	11
Normal weight	43918	29457	47	73	97	67	26	10	10**	Overweight; obesity	11
Overweight	29693	22372	50	54	96	68	35	10	9**	Obesity	9

Supplementary Figure S1. Distribution of perpetrator of workplace bullying and violence in the Work Environment and Health in Denmark study (N=17,375).

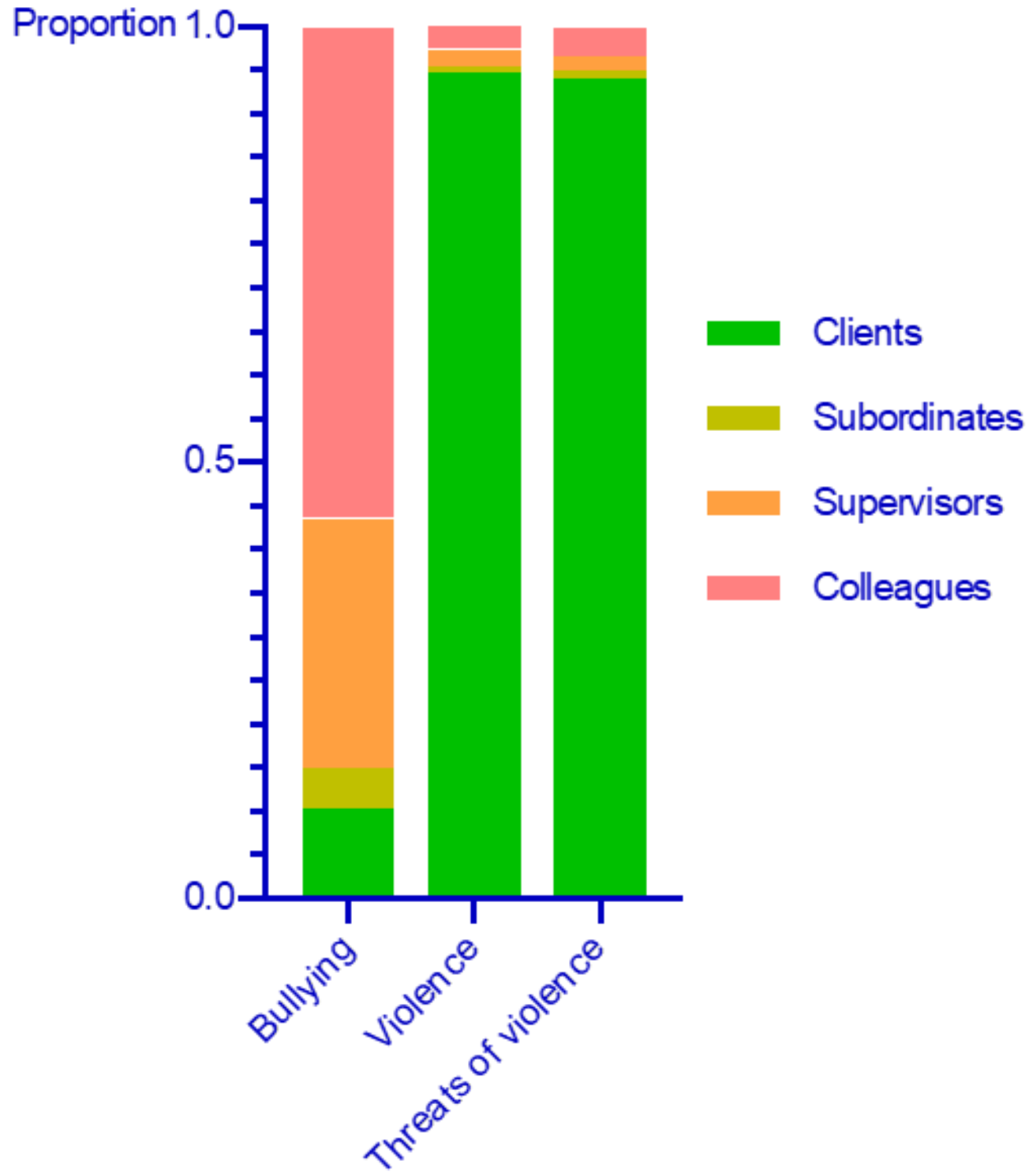

Supplementary Figure S2. Onset of workplace bullying (A and C), onset of workplace violence (B and D) and changes in categories of body mass index.

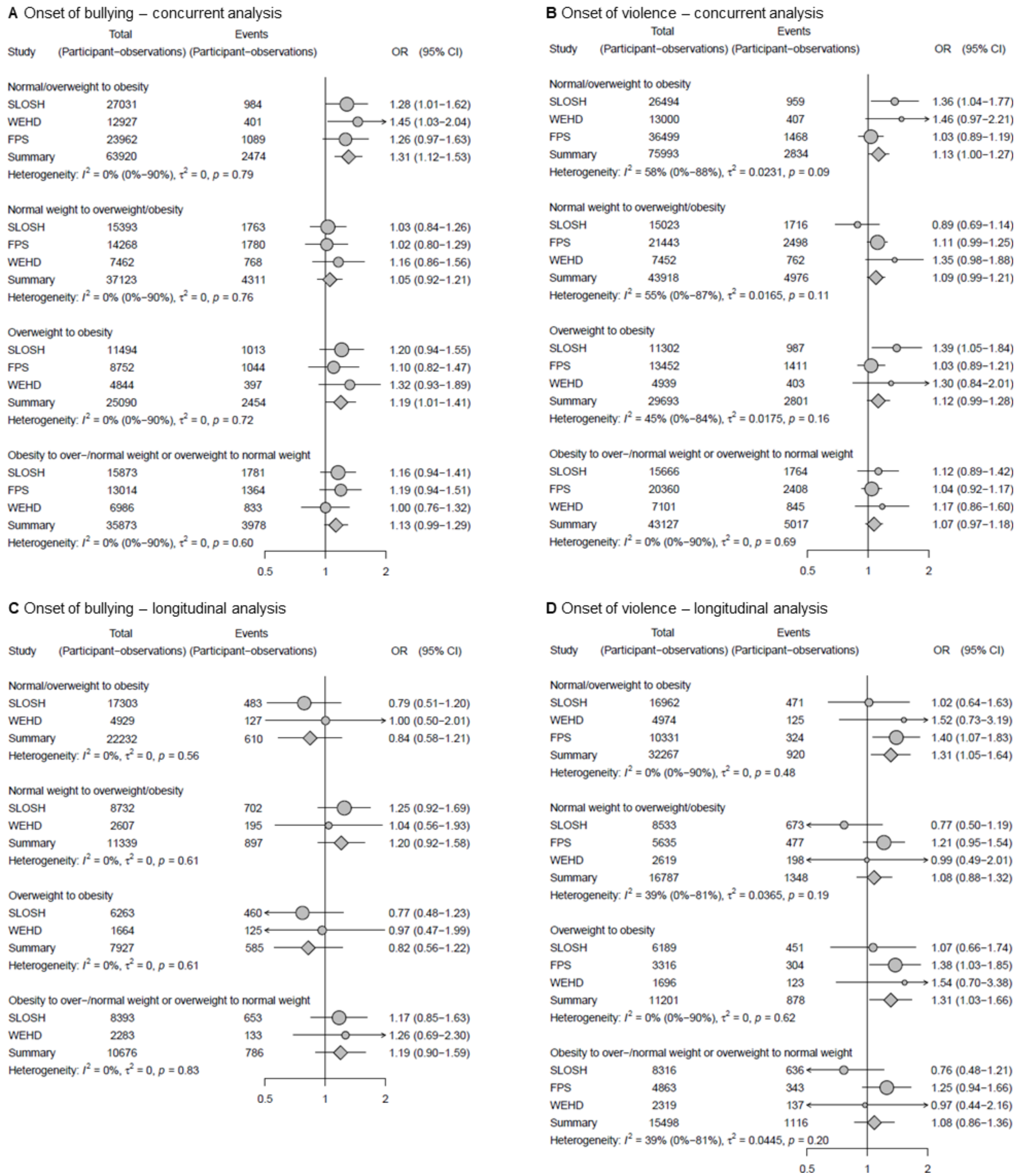

Supplementary Figure S3. Exposure-response analysis of the concurrent association between onset of workplace bullying (A), violence (B) and changes of categories of body mass index.

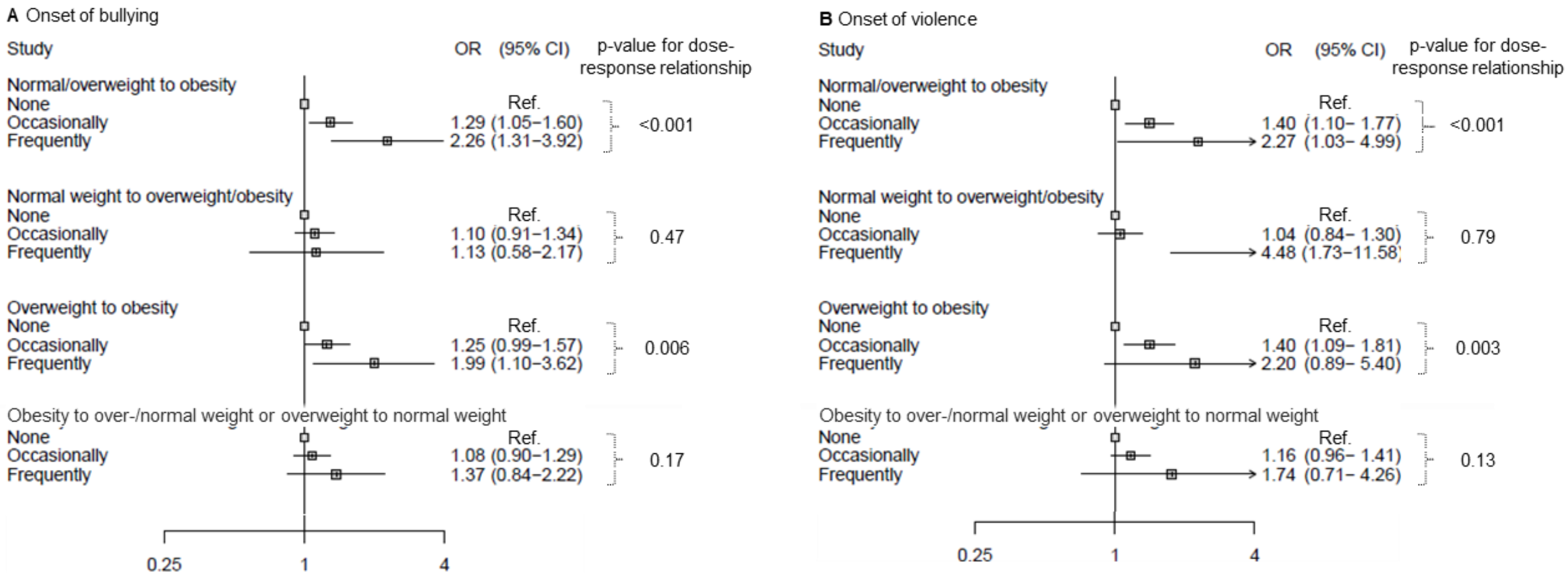

Supplementary Figure S4. Onset of workplace bullying (A), onset of workplace violence (B) and changes in health-related behaviours pooled only in data using 12 months' timeframe in the exposure measurement.

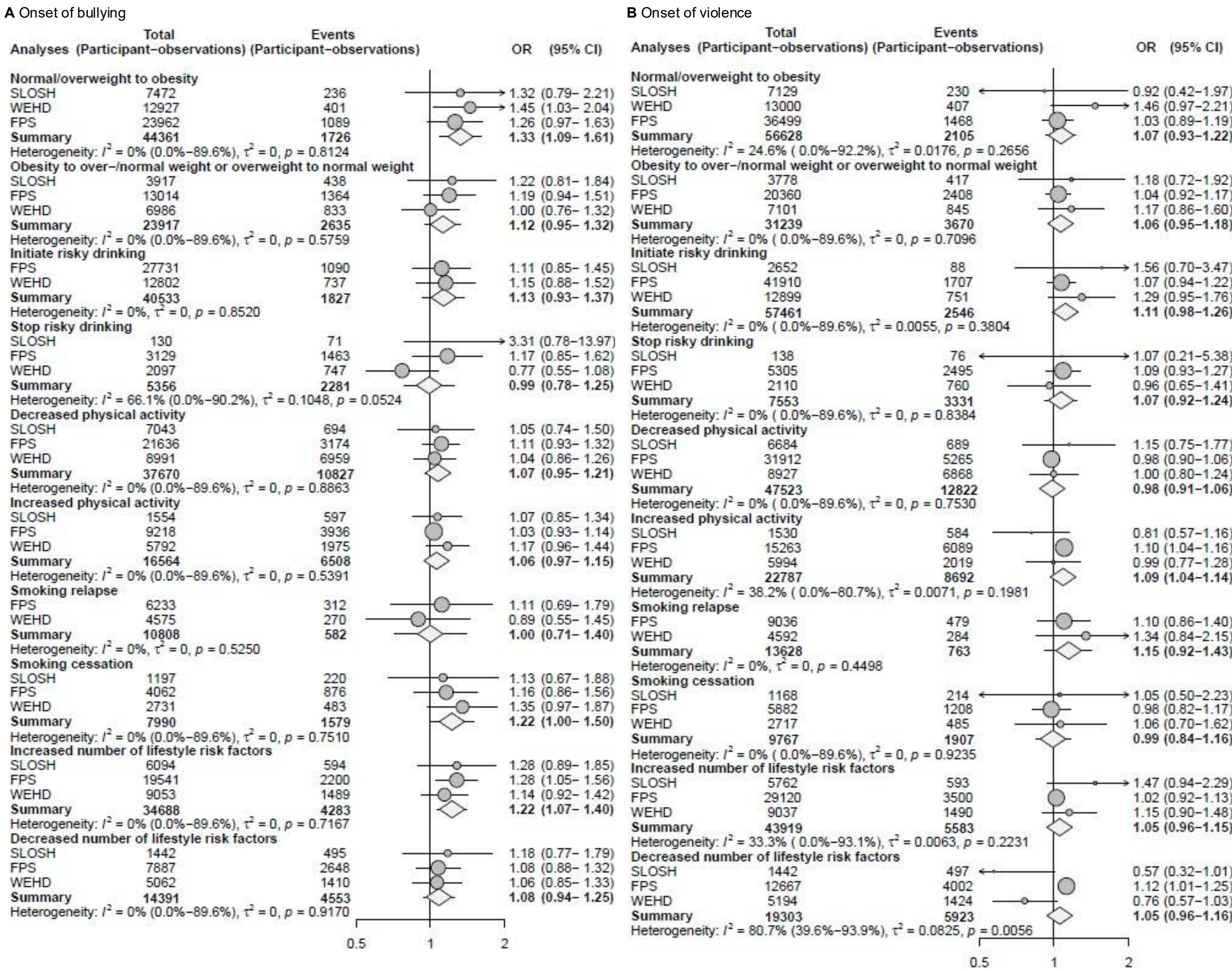

Note: SLOSH does not contain enough cases for analysis on bullying and initiating risky alcohol consumption.

Supplementary Figure S5. Onset of workplace bullying (A), onset of workplace violence (B) and changes in health-related behaviours in concurrent analysis, with additional adjustment on nightshift work.

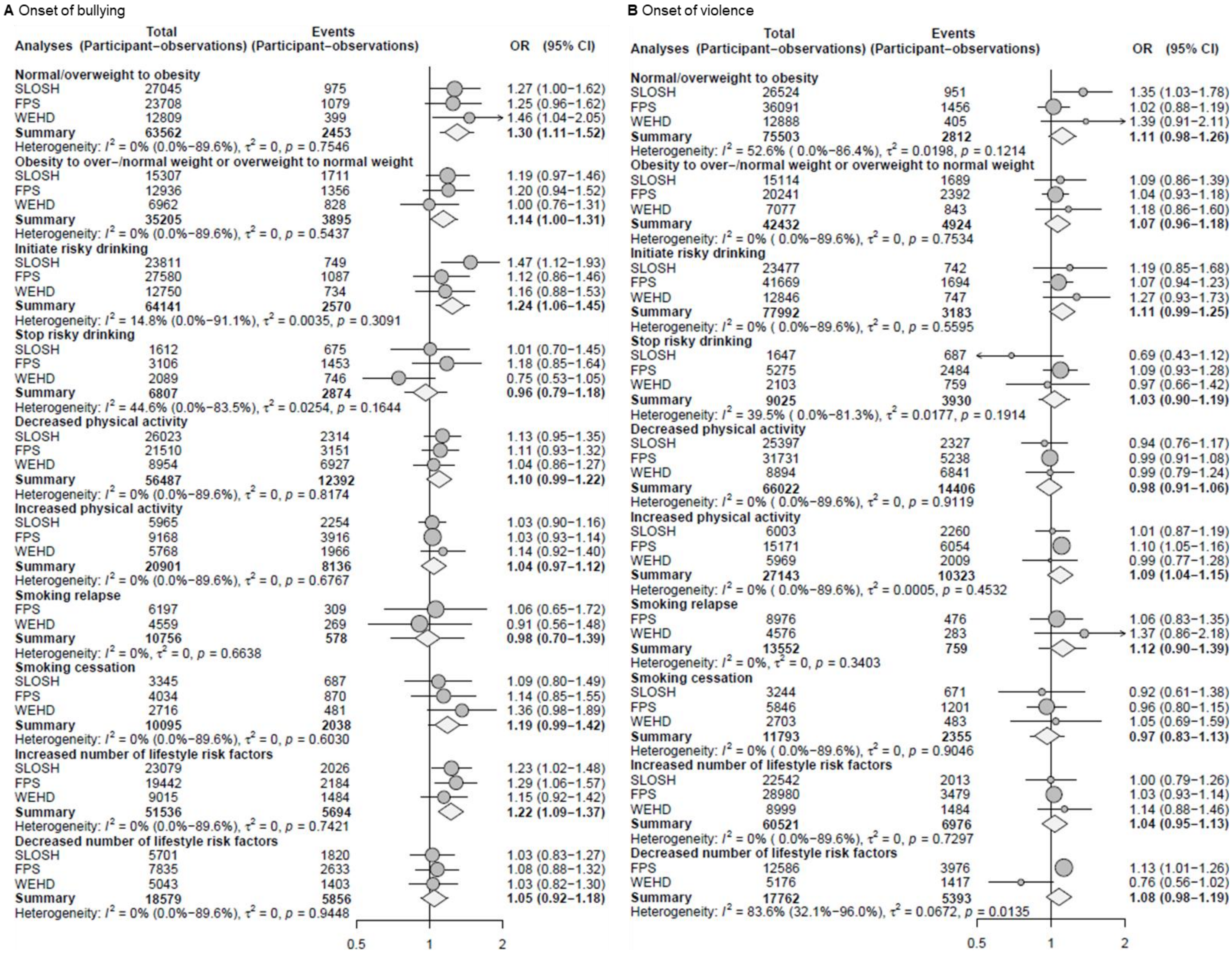

Supplementary Figure S6. Onset of workplace bullying (A), onset of workplace violence (B) and changes in health-related behaviours in concurrent analysis, with additional adjustment on nightshift work and sleep disturbances.

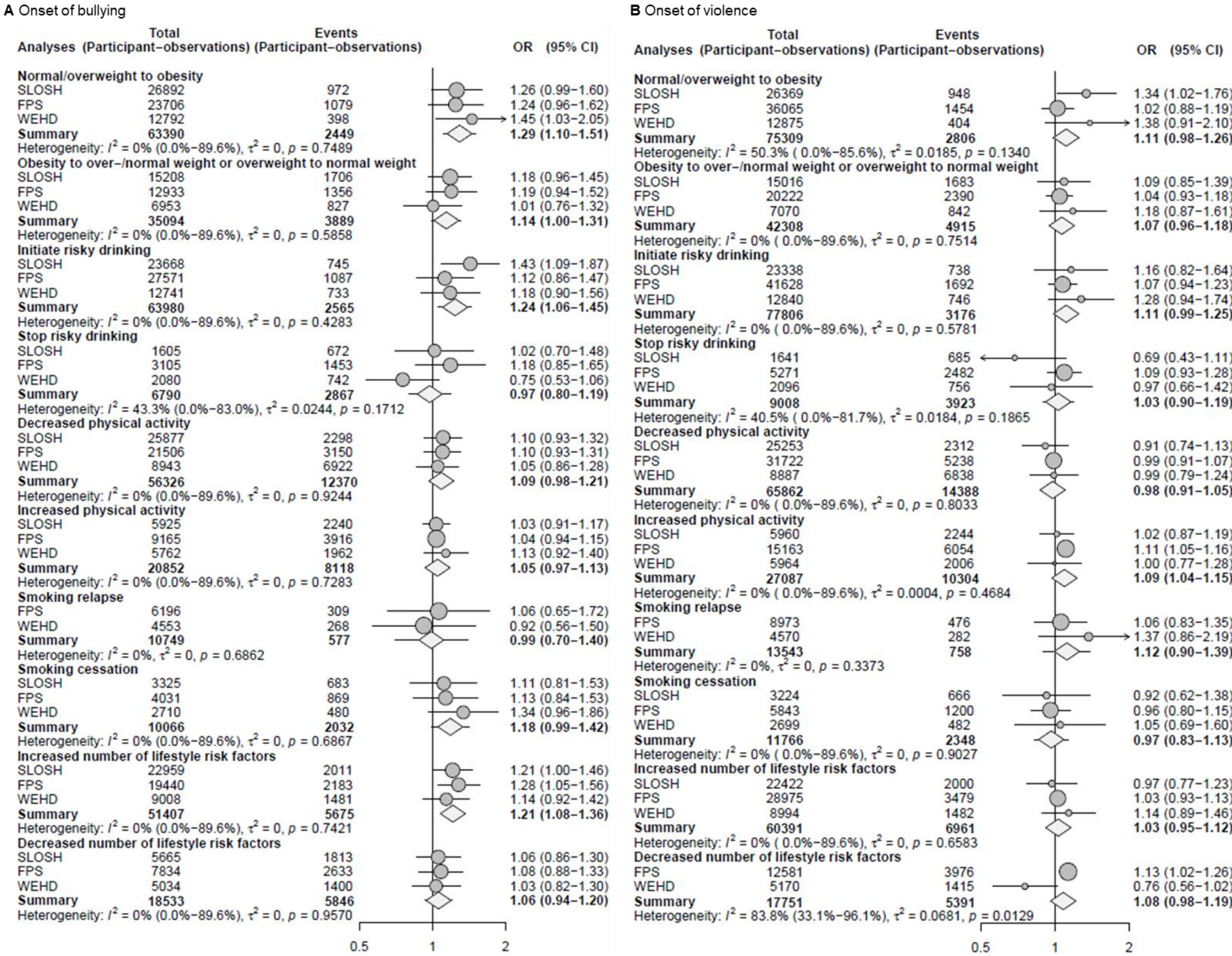

Supplementary Figure S7. Onset of workplace bullying (A), onset of workplace violence (B) and changes in health-related behaviours in longitudinal analysis, with additional adjustment on nightshift work.

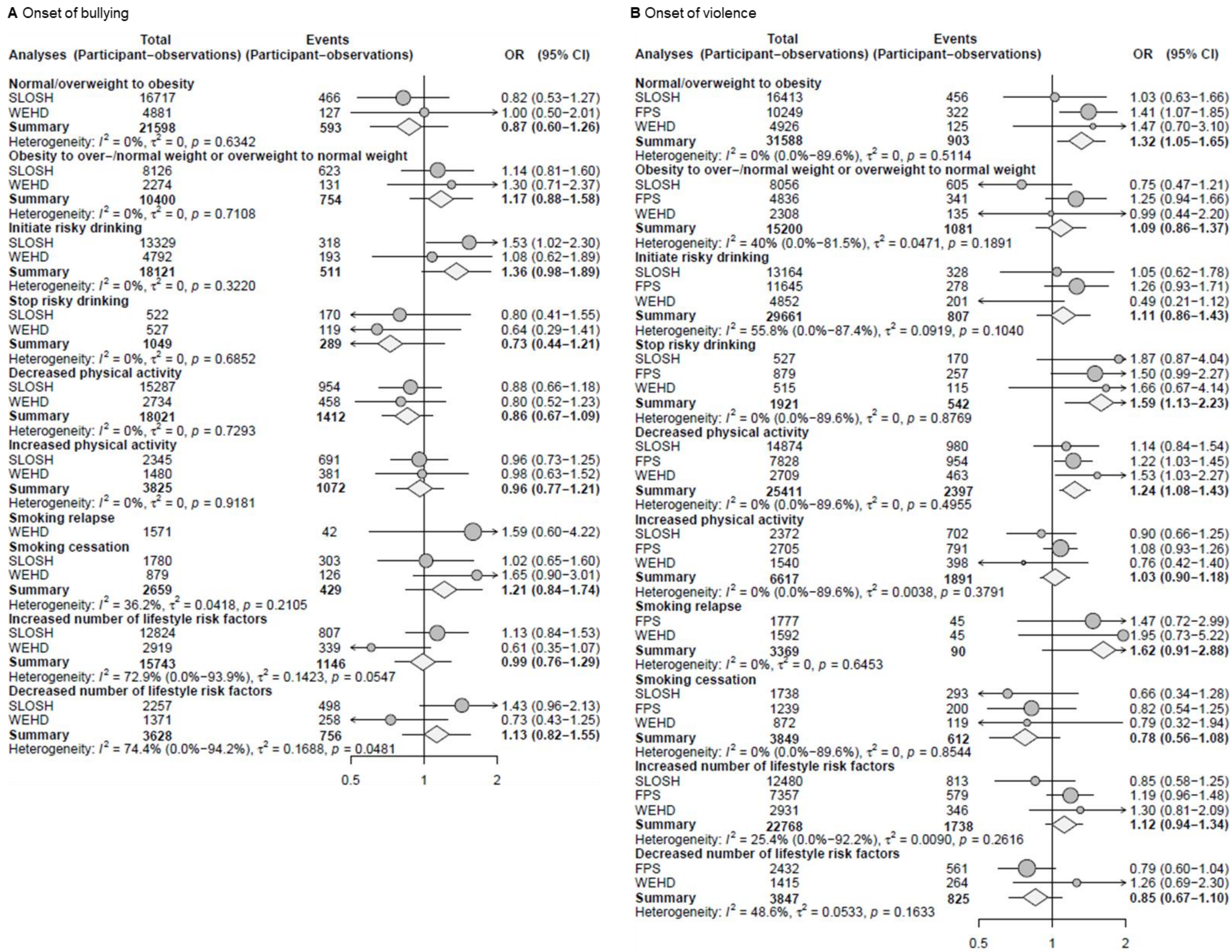

Supplementary Figure S8. Onset of workplace bullying (A), onset of workplace violence (B) and changes in health-related behaviours in longitudinal analysis, with additional adjustment on nightshift work and sleep disturbances.

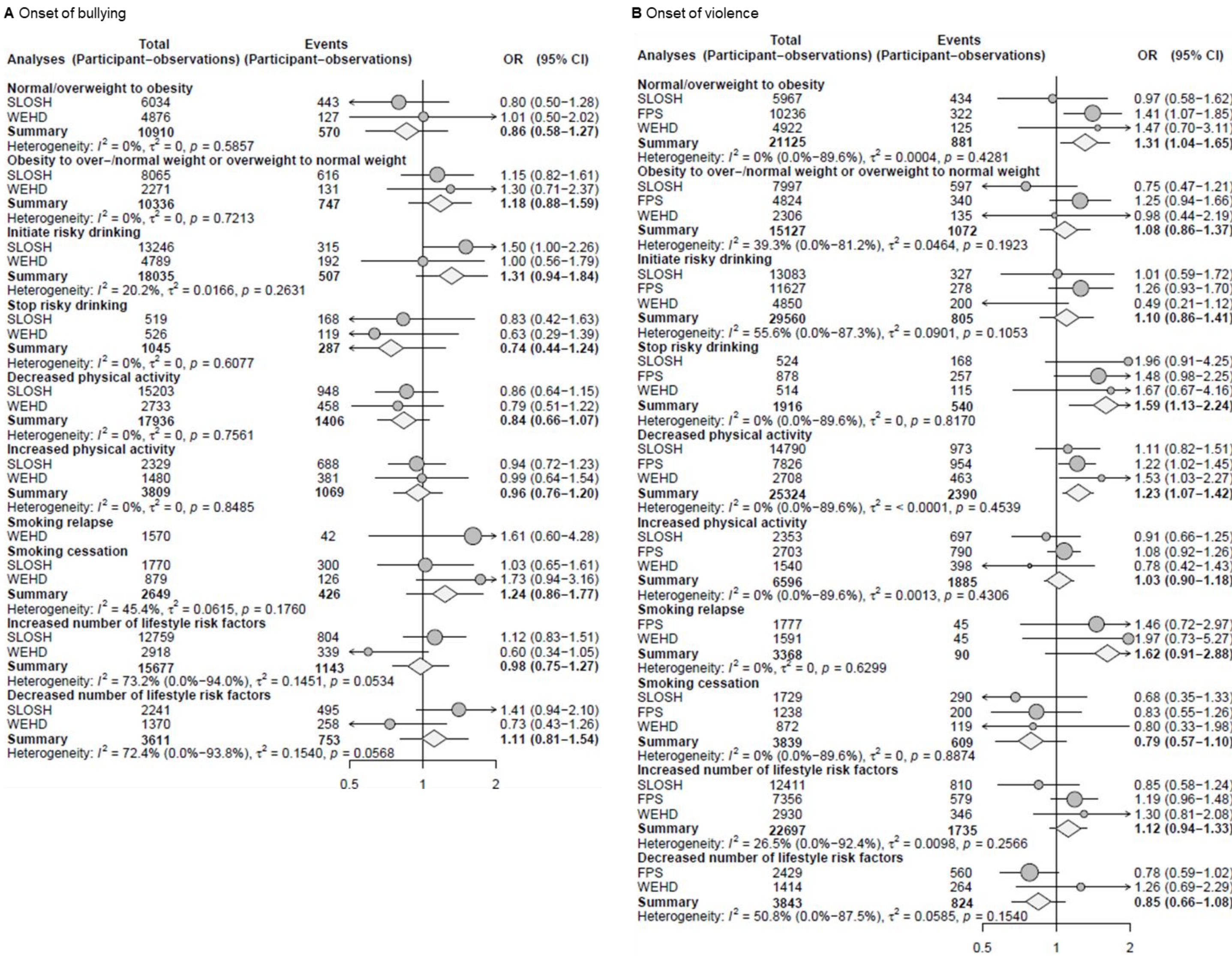

Supplementary Figure S9. Exposure-response relationship of onset of workplace bullying (A) and onset of workplace violence (B) on health-related behavioural changes in SLOSH and WEHD. None=less than once a year; Occasionally=at least once in a year; Frequently=daily; weekly., with additional adjustment on nightshift work.

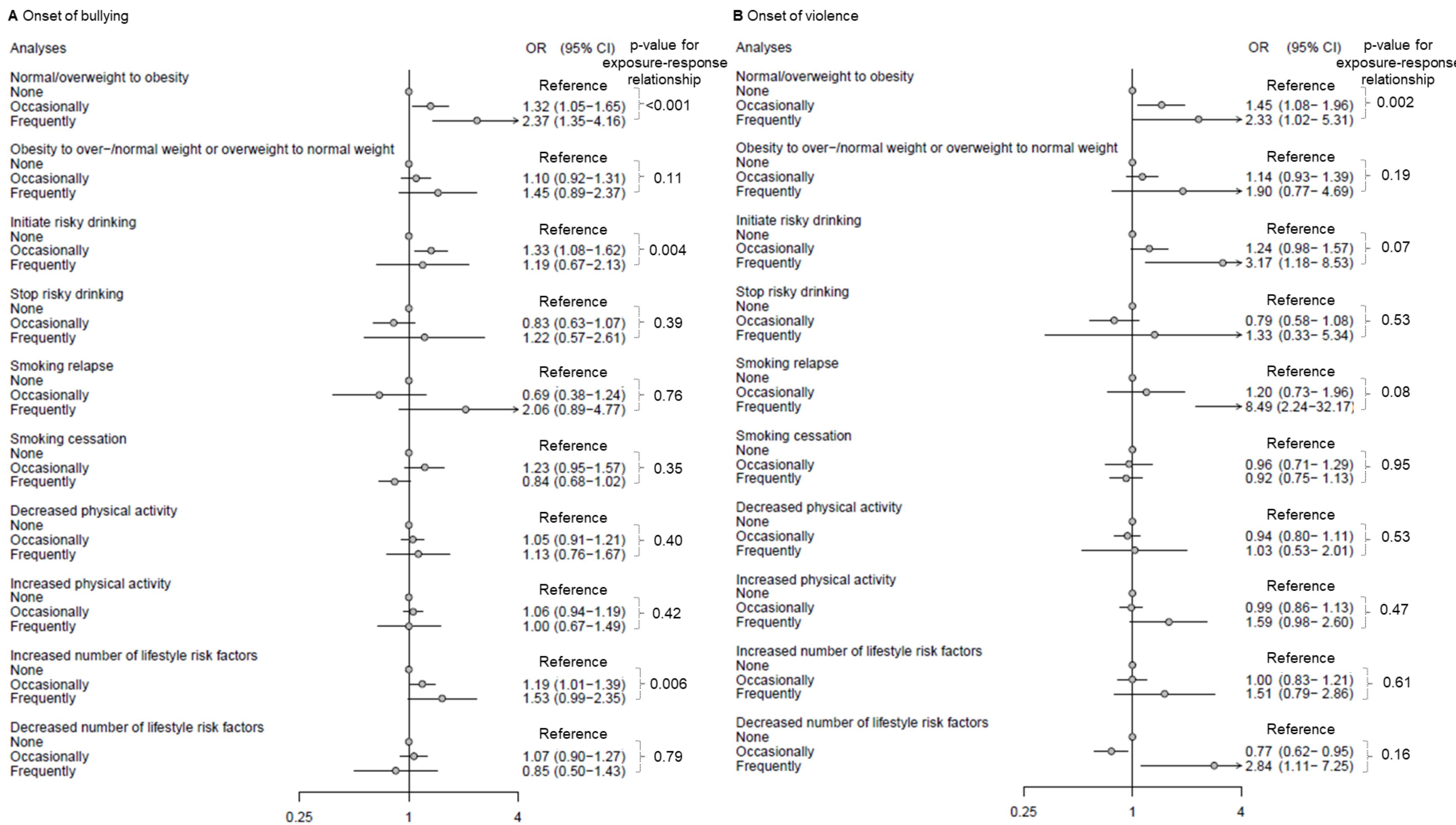

Supplementary Figure S10. Exposure-response relationship of onset of workplace bullying (A) and onset of workplace violence (B) on health-related behavioural changes in SLOSH and WEHD. None=less than once a year; Occasionally=at least once in a year; Frequently=daily; weekly., with additional adjustment on nightshift work and sleep disturbances.

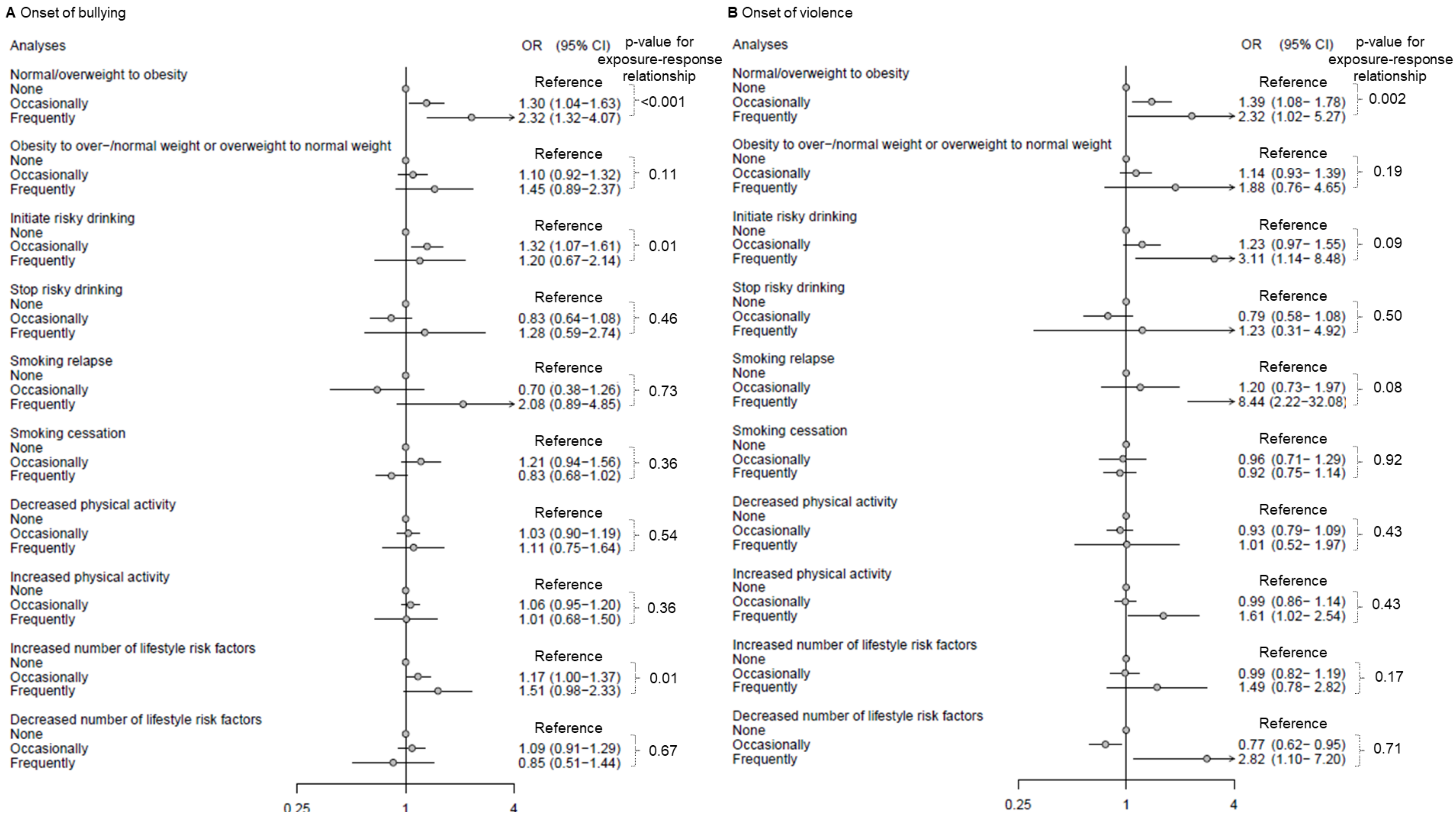

Supplementary Figure S11. Onset of workplace bullying (A), onset of workplace violence (B) and changes in health-related behaviours stratified by sex.

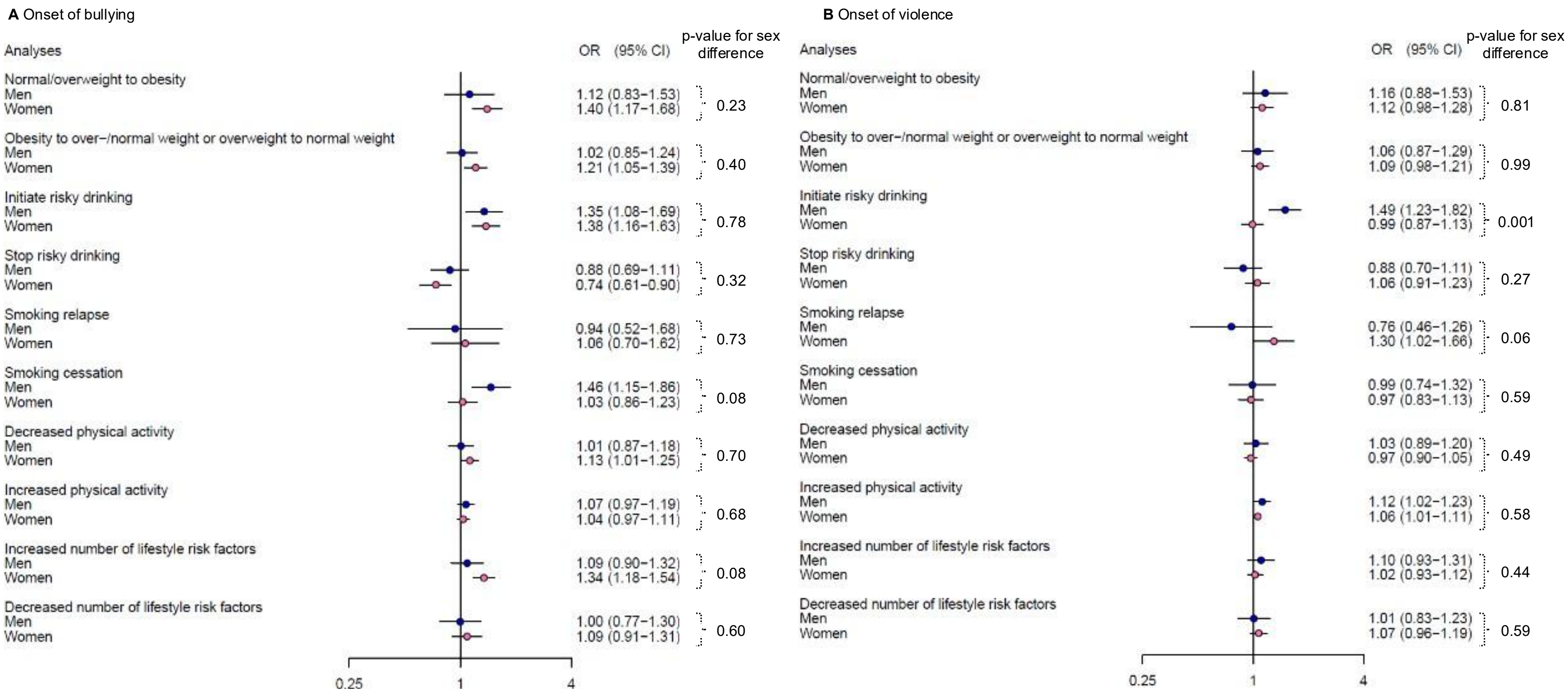

Supplement: Supplementary file 1 [file SJWEH-52-549-S001.pdf]
